# Development, Validation, and Two-Year Application of Rapid and Simple LC-MS/MS-Based Method for the Determination of K2MK-7 in Blood Samples

**DOI:** 10.3390/molecules28186523

**Published:** 2023-09-08

**Authors:** Łukasz Paprotny, Dorota Szewczak, Iryna Bryshten, Dorota Wianowska

**Affiliations:** 1 ALAB Laboratories, Research and Development Centre, ul. Ceramiczna 1, 20-150 Lublin, Polanddorota.szewczak@alab.com.pl (D.S.); 2 Department of Chromatography, Institute of Chemical Sciences, Faculty of Chemistry, Maria Curie-Skłodowska University in Lublin, Pl. Maria Curie-Skłodowska 3, 20-031 Lublin, Poland

**Keywords:** menaquinone-7 analysis, vitamin K vitamers, sample preparation, extraction, chromatographic analysis, diagnostic tool, population variability

## Abstract

Biological properties of menaquinone-7, one of the vitamin K2 vitamers (K2MK-7), both those proven and those that remain to be investigated, arouse extensive interest that goes beyond the strictly scientific framework. The most important of them is the prevention of age-related diseases, considering that we live in the times identified as the era of aging societies and many people are exposed to the vitamin K2MK-7 deficiency. Therefore, an effective analytical protocol that can be adopted as a diagnostic and preventive analytics tool is needed. Herein, a simple sample preparation method followed by the liquid chromatography-tandem mass spectrometry-based method (LC-MS/MS), was used for the selective and sensitive determination of K2MK-7 in serum samples. Under the optimized conditions, using 500 µL of serum and the same amount of *n*-hexane, the reproducibility and the accuracy were obtained in the ranges of 89–97% and 86–110%, respectively, and the limit of detection value was 0.01 ng/mL. This method was used for the routine analysis. Statistical interpretation of the data from 518 samples obtained during 2 years of practice allowed for obtaining information on the content and distribution of K2MK-7 in the Polish population, broken down by the sex and age groups.

## 1. Introduction

Vitamin K represents a family of structurally related compounds containing the 2-methyl-1,4-naphthoquinone group substituted with different hydrocarbon side chains at the C3 position. Vitamin K with a long phytyl side chain is called K1 (phylloquinone), while that with a long polypropenyl side chain is called K2 and constitutes a group of homologues known as menaquinones-n (MK-n), where “*n*” stands for a number of isoprenoid units in the side chain. Both forms are found in nature, but as K1 is found mainly in green plants and algae, K2 is synthesized by bacteria and can be found in both animal products such as meat, cheese or fermented food products, and in the human digestive tract [1]. Owing to the presence of the naphthoquinone ring, both forms exhibit numerous and more or less specific biological activities, the impact of which on the human body was underestimated not long ago [2,3]. However, only the results published at the beginning of the 21st century, proving the relationship between senile diseases and vitamin K deficiency, fully reveal its importance [2,4]. Taking into account the main health problems of the modern world and the fact that aging of the population is one of the dominant trends in the 21st century, the most important biological properties are the prevention of osteoporosis and cardiovascular diseases as well as the reduction in the risk of cancer [3,4,5,6,7]. According to [2,3,4,5,6,8] these properties are more specific to MK-7, hence the growing interest in determining this compound is observed. This interest goes beyond the strictly scientific framework, and currently, the analysis of vitamin K2MK-7 becomes an important diagnostic and preventive tool [9]. This is because the half-life of MK-7 is several days, not 1–2 h as in the case of K1, and the longer half-life means that it stays longer in the blood and is much more available to extrahepatic tissues [10]. In addition, there is a large difference in the steady-state concentration of the two forms of the vitamin, and the serum concentration of MK-7 is significantly higher than the concentration of vitamin K1 when equimolar amounts are administered to patients [11]. Considering the wide area of action of the vitamin and, among others, bioavailability issues, many people are at risk of vitamin K2MK-7 deficiency. This applies especially to people with atherosclerosis, hypercholestrolemia, diabetes, dysbiosis, celiac disease, inflammatory bowel diseases, diseases of the liver and biliary tract, people undergoing long-term oral antibiotic therapy, as well as the elderly and obese ones [1,12,13]. Allied to this, it is necessary to develop a simple analytical protocol useful in the laboratory practice that will allow for selective and sensitive monitoring of K2MK-7 levels in biofluids and at the same time accelerating sample preparation and obtaining higher laboratory throughput. This task is not easy due to low dietary intake and consequently, the low concentration of this compound in the human bloodstream [1,14].

Several techniques are applied for vitamin K determination in the biological samples. These are both indirect and direct approaches. Indirect methods, an example of which may be the measurement of one of the biochemical indicators, i.e., γ-carboxyglutamic acid in urine, allow us to assess whether the body lacks vitamin K and in no way measure the level of vitamin K, let alone the level of individual vitamin K [15]. In direct methods, migration separation techniques are used for the determination of K2MK-7. Among them, liquid chromatography (LC) with the fluorescent detection after the post-column zinc reduction to a stable hydroquinone derivative, is the most commonly applied [12,16,17]. The most recent one is LC coupled with mass spectrometry (MS) or tandem mass spectrometry (MS/MS) detection [9,15,18,19,20]. These techniques have great analytical capabilities; however, are insufficient to determine a compound at low concentration levels in a complex natural matrix [21,22,23,24]. Proper sample preparation is still needed, which will not only enable the detection of the non-fluorescent analyte, but also allow for the complete isolation of the compound from bioliquids and its concentration.

Most approaches for the determination of K2MK-7 use solid-phase extraction (SPE) and/or liquid–liquid extraction (LLE), with the most commonly used extractants being those with higher partition coefficients (*log P*), such as diethyl ether (0.9), dichloromethane (1.5), ethyl acetate (0.7), toluene (2.7), chloroform (2.3), *n*-hexane (3.9), and cyclohexane (3.4) [12,17,18,19,20,25,26]. These approaches use relatively large volumes of solvents (4–8 mL) in a single extraction step, with several steps usually being used to increase extraction efficiency [9]. These are also time-consuming procedures, usually taking several dozen minutes, which significantly reduces the throughput of the laboratory. In the literature, there are also procedures that require even 4 h to prepare a single sample [15], the implementation of which, under the conditions of laboratory diagnostics, focused on the analysis of a large number of samples per working shift, and speeding up work is difficult if not realistic. For these reasons, it is necessary to develop a quick, simple, cheap, and efficient sample preparation method that can be conducted using equipment typical of diagnostic laboratories. Another important issue is to use the smallest possible amount of biological sample needed for extraction. In addition, the principles of green analytical chemistry emphasized the need to reduce the consumption of toxic and expensive organic solvents. Hence, currently the attention is paid to the use of extraction techniques consuming minimal amounts of organic solvents. In this respect, liquid–liquid microextraction (LLME) is a modern alternative to the conventional LLE extraction technique [27].

Typically, LLME is used in the analysis of water, food, and natural product samples [25]. Despite the great application potential, this technique never received sufficient attention so far in the biomedical analysis. Therefore, the aim of this study is to optimize the extraction procedure for the efficient and selective K2MK7 isolation from the serum samples, in line with modern requirements, and its subsequent quantitative analysis using the recently developed and validated rapid LC-MS/MS-based method. As the optimized method proved to be an effective and useful diagnostic tool, it was used in our laboratory (Research and Development Centre, ALAB, Lublin, Poland) for the routine analyses for a two-year period to obtain information on the content and distribution of K2MK-7 in the Polish population by different gender and different age groups. Data were created from 518 serum samples; 52.1% of the samples were female ones and 47.9% of the male samples. This information will be useful for the subsequent development of K2MK-7 reference ranges in Poland.

## 2. Results and Discussion

### 2.1. Optimization of the Sample Preparation Procedure

**Figure 1:**
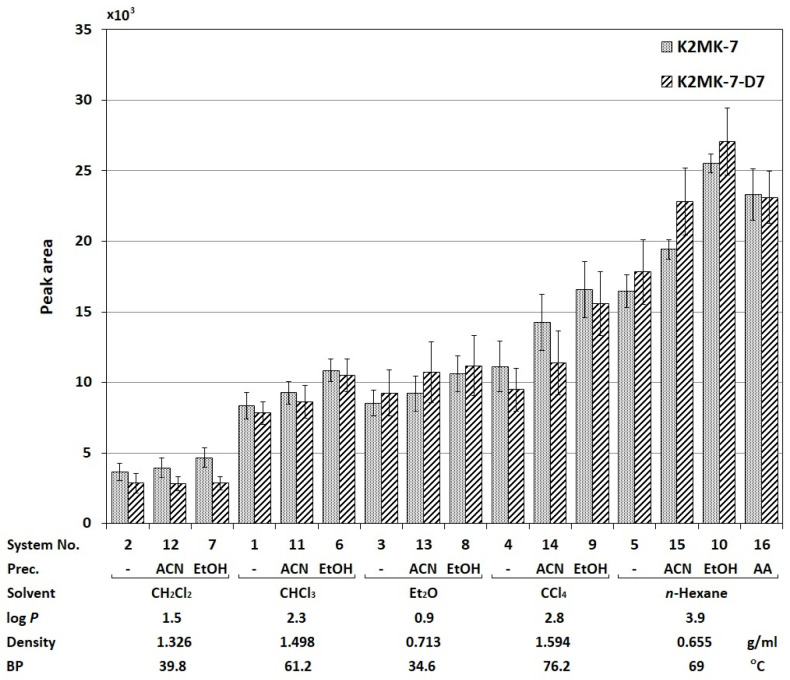
Effects of LPME variables on the recovery rates of K2MK-7 and K2MK-7-D7. Explanation of abbreviations—see the relevant section of the text above.

In this study, in order to optimize extraction procedure for the quantitative analysis of vitamin K2MK-7 in serum, 16 extraction systems were tested, differing in the type of the applied extraction solvent: dichloromethane (CH_2_Cl_2_), chloroform (CHCl_3_), diethyl ether (Et_2_O), carbon tetrachloride (CCl_4_), and *n*-hexane, its volume (250 or 500 µL), and the addition of a precipitant: acetonitrile (ACN), ethyl alcohol (EtOH), and ammonium acetate (AA). Their characteristics are presented in the Section 3. The tests were carried out using the optimized LC-MS method (see the Section 3) and discussed in the Section 2.2. The studies were carried out in the 2 mL Eppendorf vials, using 500 µL of the serum free from the test substance, fortified with the known amount of the vitamin K2MK-7 standard and K2MK-7-D7 at a concentration of 2 ng/mL. The results are presented in Figure 1 as the mean values of the peak areas of the analyte and the internal standard obtained for a given system and calculated from three independent extractions, indicating both the solvent and the factor increasing the extraction efficiency for the sake of clarity discussion. In order to assess the effect of changing the extraction conditions on the analyte peak area, the obtained data were statistically processed using the one-way analysis of variance (ANOVA), and its results are presented in Table 1. For clarity, if the calculated value of F (*F_cal_*) exceeds the table value F (*F_tab_*), this indicates a statistically significant influence of the given parameter.

As can be seen from the data presented in Figure 1, the individual systems are characterized by different isolation efficiencies of the vitamin K2MK-7 standard and its deuterated derivative. The change in the extraction solvent type differentiates clearly the size of the surface area of both compounds (*F_cal_* = 48.45, *F_tab_* = 3.48, see Table 1). In general, the smallest peak areas were observed in the extracts obtained with CH_2_Cl_2_ and the largest with *n*-hexane. At the first glance, it might seem that the observed differences are the result of different affinities of individual solvents for the analyte. However, the problem is more complex.

If the *log P* value is taken as the measure of affinity, the extraction efficiency should increase with the increase in this value. Meanwhile, the signals of both compounds observed for Et_2_O (*log P* = 0.9) are about twice higher compared to those observed for CH_2_Cl_2_ (*log P* = 1.5). Taking into account the physicochemical properties of these solvents, the possible reason for the observed differences is the lower density of Et_2_O than CH_2_Cl_2_ (see Figure 1). This fact makes it easier to collect the upper lighter ether layer manually. On this basis, it can be concluded that not only the type of solvent (its hydrophobicity), but also the method of the extraction, i.e., the ease of manipulation of the readily accessible upper layer of the extraction solvent, affects the resultant effectiveness of the compounds isolation in the miniaturized extraction systems.

Comparing the effects caused by the addition of the precipitation reagent into the extraction system (which in Figure 1 is designated as “Prec.”), it can be seen that the signals of both compounds are generally greater than those obtained previously. This is especially evident for systems with *n*-hexane and CCl_4_. This observation can be explained by achieving greater selectivity of extraction, reducing the background effect in the evaluation of the MS signals magnitude. The effect is greater for EtOH and smaller for can, which is confirmed by the values of the Fischer coefficient from Table 1, equal to 129.85 and 72.18, respectively (*F_tab_* = 3.48). However, this result contradicts the information available in the literature that ACN is a more effective precipitating agent compared to ethanol [27]. Nevertheless, according to the literature, ACN can have a negative effect on the formation of ions and can cause the so-called ion suppression phenomenon, leading to a decrease in the measured signals [28,29]. This comment applies not only to the effect of acetonitrile, but also to the other solvents used in the stage of sample preparation for analysis. The truthfulness of this statement seems to confirm the data obtained for ammonium acetate (AA). As it is commonly known, this compound increases the ionization efficiency of analytes in MS [30]. Admittedly, its use in these studies did not increase the signal magnitude of both compounds because there are no statistically significant differences in their peak intensities compared to those observed with the addition of ethanol (*F_cal_* = 3.93, *F_tab_* = 7.71, see Table 1), but it improved the precision of the signal evaluation significantly (compare the size of the bars in Figure 1).

As for the effect of the volume of the used extractant on the analyte signal, statistically significant differences (see Table 1) were observed only for the *n*-hexane extract, i.e., the solvent showing the largest peak areas of both K2MK-7 and IS. For this extractant, higher signals were observed for a smaller volume of *n*-hexane, i.e., 250 µL. Nevertheless, taking into account the complexity of the biological sample matrix and the assumed low vitamin content in real samples, the use of a smaller volume of the solvent in a single extraction cycle may result in deterioration in the measurement accuracy. The conducted studies on the impact of the number of extraction cycles on the effectiveness of K2MK-7 isolation using 500 and 250 µL of *n*-hexane (data are shown in the Supplementary Materials), showing the need for three extraction cycles for a smaller volume of extractant to achieve comparable accuracy, confirmed the validity of this reasoning. Therefore, it was decided to use 500 µL of *n*-hexane and the same amount of biological material for the final determination. There are also economic reasons behind this choice, as the use of a larger amount of solvent in a single extraction cycle allows for significant speeding up of the sample preparation and for obtaining larger throughput of the laboratory.

### 2.2. Method Validation

The proposed analytical protocol for the analysis of K2MK-7 in the human serum was successfully validated following the criteria of FDA for the bioanalytical method validation (see the Section 3) [31].

**Figure 2:**
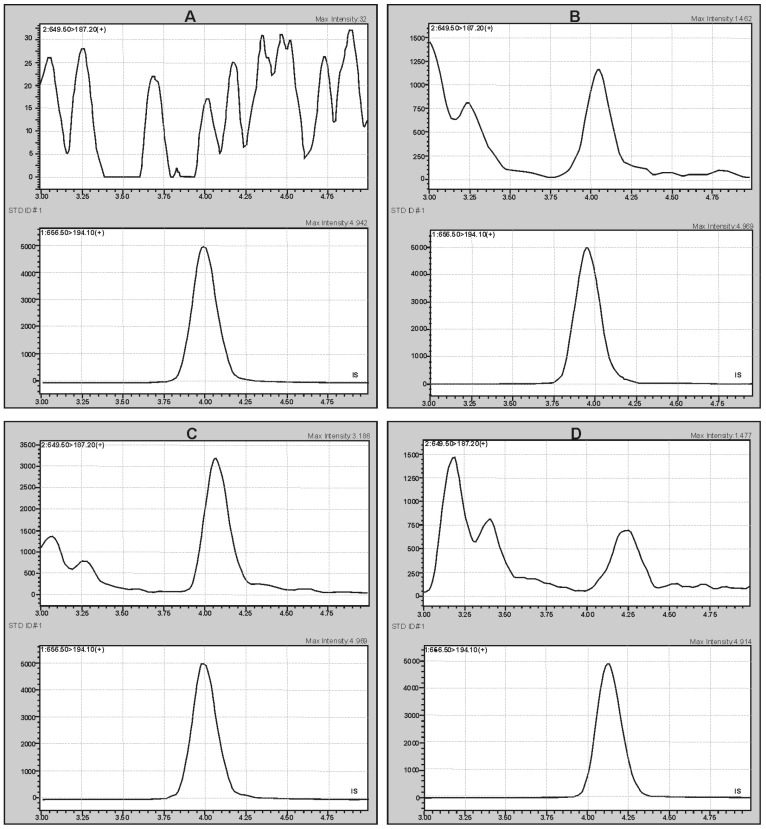
MRM chromatograms of the blank serum sample (**A**), the control sample obtained by spiking the blank serum sample with the analyte at the level of 0.35 ng/mL (**B**) and 0.96 ng/mL (**C**), and the chromatogram of the authentic serum sample tested for the content of vitamin K2MK-7 (**D**).

**Figure 3:**
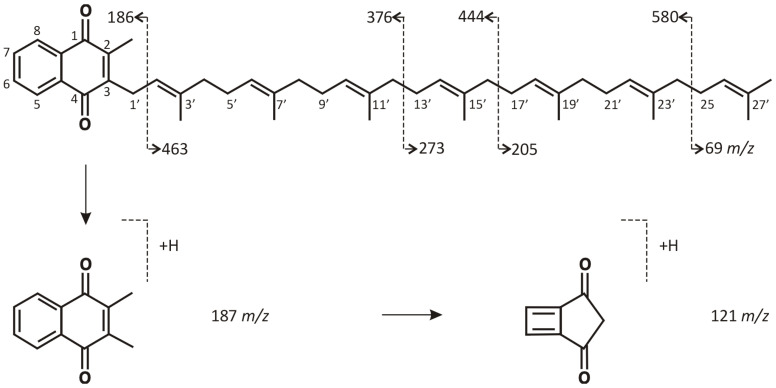
Chemical structure and a characteristic fragmentation pattern of K2MK-7 together with the chemical structure of the product ions used for the analysis.

Identification and quantification were based on the MS/MS multiple reaction monitoring (MRM) mode after studying the fragmentation spectra of the analyte and IS in accordance with the confirmation criteria taken from Commission Decision 2002/657/EC. According to them, the MRM measurements for the analytes were performed for two transitions: *m*/*z* + 649.5 → 121.0 (qualifier transition, S2) and 649.5 → 187.2 (quantifier transition, S1) for K2MK-7, and one transition *m*/*z* + 656.0 → 194.1 for K2MK-7-D7 using the collision energy (CE) values at 41 eV, 30 eV, and 37 eV, respectively (the dwell time of 500 ms). During the optimization experiments, the suitability of the ESI and APCI ionization sources was checked. Although both gave the same fragmentation ions, the intensity of the signals was higher using the APCI source, which determined the use of this ionization source. Figure 2 shows the representative APCI(+)-MRM chromatograms obtained during the validation experiments for the pooled serum samples irradiated with UV light to destroy endogenous vitamin as well as the authentic serum sample, confirming acceptable analytical conditions for both qualitative and quantitative analyses of K2MK-7 in the serum samples. Figure 2A shows the chromatograms of the blank serum sample (upper chromatogram) spiked with deuterium labeled K2MK-7 (K2MK-7-D7) used as the internal standard (lower chromatogram, IS). Figure 2B,C shows the chromatograms obtained for the pooled serum samples with the addition of IS and the analyte at the level of 0.35 ng/mL and 0.96 ng/mL, respectively. Figure 2D shows the chromatograms obtained for an authentic serum sample tested for the content of vitamin K2MK-7. The chemical structure and possible fragmentation pattern of K2MK-7, together with the chemical structure of the product ions used for the analysis, are presented in Figure 3.

The specificity of the method was confirmed by matching the retention times (RT) and the fragmentation ion signal ratio (S2/S1) obtained for the analyte in five different aliquots of the authentic serum samples to the mean values obtained for the calibration solutions (RT_ref_ = 4.01 min ± 2.0% and S2/S1_ref_ = 0.439) with the differences in the retention times at the −0.069 level, while the relative signal difference was −1.82%.

The calibration curve was constructed not using the weighted model, which resulted in small and evenly distributed residual errors. The curve is shown in in the Supplementary Materials. Its characteristic features determined by the slope, the intercept (±confidence interval at the confidence level of 0.95), and coefficient of determination (R^2^) are 1.30621 (±0.106), −0.00789 (±0.0905), and 0.991, respectively. The *F*-value obtained for the statistical method of the lack of fit was equal to 0.79 (*F_cal_*), with the values of the numerator and the denominator 7 and 18, respectively, and the tabular *F*-value equal to 2.58 (*F_tab_*). *F_cal_* < *F_tab_* confirms the linear relationship between the ratio of K2MK-7 to IS signals and the K2MK-7 concentration. The back-calculation of K2MK-7 concentrations in the calibration solutions, performed to confirm suitability of the used calibration, showed acceptable deviation of the interpolated concentrations of standards. Inaccuracy and imprecision of K2MK-7 determinations, expressed as % bias and % CV, were in the range of −11.0–5% and 2.3–14.2% for six out of seven non-zero calibration solutions. Only in the case of the smallest standard concentration was the deviation at 18% obtained, and this concentration was accepted as the LLOQ level.

Determination of the signal-to noise ratio performed using the LLOQ sample to establish the smallest analyte content allowing to confirm its presence (LOD) and to quantify it (LOQ) reached 0.013 and 0.039 ng/mL.

The data on the inaccuracy and imprecision of the present study are presented in Table 2. The intra-and inter-day inaccuracy, expressed in % bias, ranged from −13.8 to 10.1% with the precision of 3.3–11% (% CV). These results show adequate great precision and accuracy. The correctness of this statement confirms the ANOVA and the Student’s *t*-test results showing that there is a lack of a significant difference between the results at the individual concentration levels (1.00 < *F* > 3.22, *F_tab_* = 3.48; −0.06 < *t_cal_* < 2.46, *t_crit_* = 4.30). The dilution study carried out at 1:1, 1:8, and 1:16 for the samples fortified above the highest concentration of the calibration standard showed that the dilution can be undertaken with a sufficiently good precision and accuracy. The precision of the study ranged from 6.4 to 12.8% CV and the accuracy ranged between −10.5 and 8.2% bias.

Various storage and handling conditions were evaluated to determine their effect on the vitamin K2MK-7 stability. The obtained results are given in Table 2. The samples kept in darkness in the autosampler, refrigerator, and freezer at a temperature from 15 to −18 °C for 24 h are stable. Alternatively, the samples exposed to light at room temperature show a loss exceeding the accepted stability criterion after just one hour [25,32]. The stability study after four freeze–thaw cycles showed that the analyte is stable up to three cycles. These data prove that, while the low temperature under the tested conditions stabilizes K2MK-7, one should avoid definitely exposing the sample to UV radiation. Moreover, multiple freezing and thawing of serum samples subjected to the K2MK-7 determination should be avoided.

Table 3 summarizes the results of the matrix effect (ME), recovery (RE), and process efficiency (PE) studies (for details, see the Section 3). These parameters were obtained using the method described in [33] by determining the area of the K2MK-7 and IS peaks for three differently prepared sets of samples, each at three concentration levels. The parameters are calculated as a percentage of the response of set 2 samples in relation to those of set 1 samples (ME), the response of set 3 samples in relation to that of set 2 (RE), and the response of set 3 samples in relation to that of set 1 samples (PE) [33]. The obtained values confirm a slight increase in the ionization of the analyte by co-eluting substances from the biological matrix. This effect is especially pronounced at smaller analyte concentrations. Nevertheless, the efficiency of the process is comparable and high. The recovery ranges from 79 to 85% and from 84 to 88% for the analyte and IS, respectively. These values are suitable for the quantitative K2MK-7 analysis in the human serum.

### 2.3. Practical Application

To assess the applicability of the optimized method for the routine laboratory practice, studies were carried out in the group of 10 healthy volunteers with normal dietary habits. The research confirmed that the method is suitable for the analysis of vitamin K2MK-7 in the real serum samples. Therefore, after carrying out comparative tests showing compliance of the results at the level of 85–90% with the other laboratory tests (see the Supplementary Materials), the decision was made to implement the developed method as an analysis routinely performed in our laboratory. The results presented below constitute the statistical interpretation of the data obtained during the standard practice of our laboratory during the 2-year period. As mentioned, these data were obtained from 518 serum samples; 52.1% of the samples were female ones and 47.9% were male samples. The K2MK-7 levels in the individual age ranges: <10, 11–20, 21–30, 31–40, 41–50, 51–60, 61–70, and >70 were created based on the analysis of the following number of female (male) samples: 14 (12), 20 (26), 42 (37), 60 (54), 54 (37), 38 (53), 32 (19), and 10 (10). These data are important because, to our knowledge, these are the first available literature data on the content of vitamin K2MK-7 in the Polish community. As a result of the life expectancy extension, as well as changes in the age structure of the population, the median age of Poles as well as of other nationalities is growing year by year. According to WoldData.info [34], among 119 countries assessed in 2018–2020, Poland ranks 28th with the average age of 41.9, which means that half of the Polish population is older than 41.9. Japan is in the lead in this ranking, with the average age of 48.6 years. The USA, for comparison, ranks 44th with the average age of 38.5 years.

**Figure 4:**
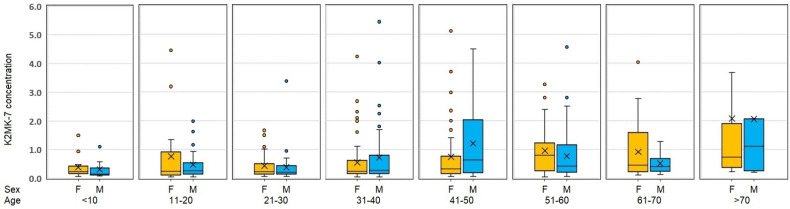
Box plot of the serum concentration of K2MK-7 for different age groups and gender.

The graphical representation of the obtained data is shown in Figure 4 in the form of box plots with the division into the sex- and age-related groups. Their analysis allows us to conclude that, in line with the expectations, the content of vitamin K in the Polish society is generally small. However, as can be seen in the studied age groups for individual genders, the obtained field ranges, their position, median values (marked as the line across the box), and average values (marked with the cross) are different. These features independently confirm the applicability of the developed method for the routine analysis of vitamin K2MK-7 content in the serum samples.

The more detailed analysis of the presented data shows that an asymmetric distribution of values is observed in each group. With age, the width of the box ranges for both genders increases, which means that the degree of results dispersion increases. The highest degree of agreement of the results with the lowest number of outliers and the shortest whiskers is visible for the youngest age group, with the mean and median values in the female and male groups being 0.319 and 0.154 ng/mL as well as 0.387 and 0.239 ng/mL, respectively. In turn, the lowest degree of agreement with the longest whiskers can be observed in the group of males in the middle age group, i.e., 41–50 years old, with the highest values of outliers, not shown in Figure 4 for the sake of legibility, obtained in the oldest age group of females (11.7 ng/mL). In this age group, the highest mean and median values for female and male are also noticeable. These values are 2.14 and 0.73 ng/mL for female and 1.98 and 1.18 ng/mL for male. This observation is consistent with the general knowledge about the increasing incidence of cardiovascular diseases with age and the effect of blood thinning drugs such as warfarin on the content of vitamin K in the body [5,13,35]. Moreover, the smaller content of vitamin K2MK-7 in females compared to its content in males is consistent with the greater frequency of osteoporosis in females [12]. The difference in the amount of vitamin K2MK-7 in females and males is noticeable in the age group 41–50, which is when menopause often begins. These observations, as well as other median values in the figure, visible in the form of a different position of the line in the box, may suggest the probability of a relationship of age and gender with the vitamin K content in the human body. This suggestion was verified by the statistical analysis, the results of which are collected in Table 1, together with those of the statistical analysis of factors influencing the stage of sample preparation. The obtained Spearman’s rank correlation coefficients (*rho value*, see Table 1) revealed that the K2MK-7 values are significantly associated with the age both in females (r = 0.23, *p* < 0.001) and males (0.27, *p* < 0.001). Higher values of the *F*-test obtained for females (*F_cal_* = 4.18, *p* < 0.001) not only confirmed the relationship between the age and gender with the content of vitamin K, but also indicated that in the female group, there is a greater distribution of K2MK-7 values depending on age.

### 2.4. Comparison with the Published Data

Due to the great interest in the role of vitamin K2MK-7 in the human body, and consequently, the sensitive analysis of this compound in the physiological fluids, a number of different approaches to its direct determination can now be found in the literature, including the LC-MS/MS method [12,13,15,16,17,18]. The disadvantages of most of these methods are the consumption of large amounts of both sample and other organic solvents, quite complicated equipment used at the sample preparation stage, long analysis time, and high LOD values. Riphagen et al. [19] published the LC-MS method with APCI for determination of vitamins K1, K2MK-4, and K2MK-7 with the simplified pretreatment sample procedure but the limit of quantification for K2MK-7 was only 2.2 ng/mL. This value is definitely not suitable for monitoring the vitamin levels in the healthy population, and even less so for assessing its deficiency. On the other hand, Dunovska et al. [36] proposed the method with the LOD value of 0.002 ng/mL, but with the sample preparation procedure taking up to 4 h, which excludes the possibility of its use in the diagnostic laboratory. Our method definitely stands out because by combining the simplicity of the miniaturized LLE technique with the possibilities of the LC-MS/MS method, we obtained a fast and cheap method that requires a 0.5 mL serum sample and only 0.5 mL extractant to guarantee the high analyte recovery with the small LOD value in about 5 min, with the total analytical run time of 10 min needed to elute the more hydrophobic interfering compounds. Justice should be given, as due to the low content of K2MK-7 in real patient samples, we were unable to reduce the volume of serum needed for extraction in a single extraction run. Nevertheless, although the volume proposed in our methodology is larger than the 350 µL used by Riphagen, it is analogous to the volume used by Dunovska, with a total of 8 mL of solvent used for extraction.

The resulting significant shortening of the overall analysis time, with a radical reduction in the exposure of the unstable analyte to stress factors, made it possible to notice subtle relationships between the age and the gender and the content of vitamin K. These dependencies were not noticed by Dunovska et al. [36], who reported the content of vitamin K2MK-7 in the population of Caucasian as 0.074–0.759 ng/mL for both females and males.

## 3. Materials and Methods

### 3.1. Sample Preparation

Methanol (MeOH), ethanol (EtOH), and acetonitrile (ACN), all of LC/MS grade, were purchased from Merck (Darmstadt, Germany). Carbon tetrachloride (CCl_4_), chloroform (CHCl_3_), diethyl ether (Et_2_O), methylene chloride (CH_2_Cl_2_), *n*-hexane, and ammonium acetate (AA) (all with analytical purity grade) were purchased from the Polish Chemicals Plant POCh (Gliwice, Poland), and 99% formic acid of LC-MS purity was from VWR Chemicals (Gdańsk, Poland). The certified standards of K2MK-7 and deuterium-labeled K2MK-7 (K2MK-7-D7) were obtained from Sigma-Aldrich (St. Louis, MO, USA). The first standard was delivered as a solution in ACN at the concentration of 100 µg/mL. K2MK-7-D7 used as the internal standard (IS) was in the powder form in the batch of 1 mg. It was dissolved in 1.5 mL of EtOH as recommended by the supplier.

Stock solutions of standards were prepared in EtOH by diluting the K2MK-7 standard solution to the concentrations of 10 and 100 ng/mL and the IS standard solution to 50 ng/mL. These solutions were used for calibration, validation, and analysis of real samples. They were kept under the stable conditions at −20 °C (±2 °C) until their use. All other solutions applied for determining validation parameters were prepared on the ongoing basis, unless otherwise stated, from the individual stock standard solutions, being diluted in water and/or in aliquots of the pooled blank serum sample under the conditions protecting them against light.

The quality control (QC) samples were prepared in the pooled serum sample spiked with the individual stock standard solutions. To avoid multiple thawing, these samples were thoroughly mixed to ensure homogeneity, then divided into smaller portions that were separately frozen and stored at −20 °C (±2 °C) until needed.

All samples used in the study were kept in the amber nontransparent vials unless stated otherwise. Water was purified using the Milli-Q system (Millipore Sigma, Bedford, MA, USA).

### 3.2. Collection and Storage of Serum Samples

During the assessment of the suitability of the developed method for the routine laboratory practice, the samples for testing were obtained from 10 healthy volunteers with normal dietary habits after an overnight fasting period. Small volumes of blood samples were collected by the qualified staff in accordance with the local, national, and international regulations (with the Declaration of Helsinki) on ethics after obtaining the informed consent from each of them. Blood was obtained by venipuncture into the 4.9 mL tubes using a single closed system containing the Monovette coagulation activator according to the manufacturer’s instructions (Sarstedt AG, Nümbrecht, Germany). Then, it was thoroughly mixed in order to maintain its homogeneity and immediately protected against light using aluminum foil. The serum was separated within 45 min of blood collection by centrifugation (2000× *g*; 10 min at room temp.) and frozen at −20 °C until testing.

Age- and gender-specific K2MK-7 intervals in the Polish population were created using the data obtained from the samples collected during our laboratory standard practices (Research and Development Center, Alab Laboratories, Lublin, Poland). Blood was collected from the patients only during their laboratory tests (no additional material was collected).

### 3.3. Sample Preparation

The optimized procedure consists of mixing 500 µL of the serum sample mechanically with 5 µL of the internal standard solution and 245 µL of EtOH in the 2 mL amber Eppendorf vial, then adding 500 µL of *n*-hexane, 5 min vortexing, and finally centrifugation (14,500 rpm, 2 min). Then, the supernatant upper layer is quantitatively transferred to the dark glass vial, evaporated to dryness under a stream of nitrogen, then reconstituted in 50 µL of methanol with 0.1% formic acid, and after transfer to the insert vials, analyzed.

In order to determine the optimal extraction conditions for the quantitative determination of K2MK-7 in serum, the influence of the following factors on the analyte and IS peak signals was investigated: the type of the extraction solvent, the ratio of volumes of the extraction solvent to the sample, and the addition of a precipitating reagent. The experiments were conducted in 16 systems. Their characteristics are given below.

In systems 1–5 to 500 µL of serum, 500 µL of the suitable solvent were added, i.e., CHCl_3_ (system 1), CH_2_Cl_2_ (system 2), Et_2_O (3), CCl_4_ (4), and *n*-hexane (5). In systems 6–10 to 500 µ of serum and 250 µ of the solvent, i.e., CHCl_3_ (in system 6), CH_2_Cl_2_ (7), Et_2_O (8), CCl4 (9), n-hexane (10), and also 250 µL of EtOH were added each time. In systems 11–15 to 500 µL of serum and 250 µL of the solvent, i.e., CHCl_3_ (in system 11) or CH_2_Cl_2_ (12) or Et_2_O (13) or, CCl_4_ (14) or *n*-hexane (15), and 250 µL of ACN were added each time. In system 16, as before, 500 µL of serum and 250 µL of *n*-hexane were used with the addition of 100 µL of 1 M ammonium acetate (AA) solution.

For the system characterized by the highest intensity of analyte and IS signals, the effect of the number of extraction cycles was also examined. The influence of 3 extraction cycles on the K2MK-7 extraction efficiency was taken into account. In all these experiments, a pooled serum was spiked with the analyte and IS at 2 ng/mL. For each of the tested systems, three independent repetitions were made (n = 3).

### 3.4. LC-APCI-MS Analysis and Its Optimization

The LC analyses were performed using the Shimadzu NEXERA X2 LC system (Shimadzu, Kyoto, Japan) composed of a parallel double binary pump (LC-30AD), a system controller (CBM-20A), an automatic solvent degasser (DGU-20A5R), and an autosampler (SIL-30AC). The autosampler temperature was maintained at 15 °C. Separations were made using the Kinetex C18 column (50 × 2.1 mm, 2.6 µm, Phenomenex, Inc., Torrance, CA, USA) applying gradient elution with the mobile phase flow at 0.6 mL/min. The mobile phase A was the solution of 0.1% formic acid in water; the mobile phase B was 0.1% formic acid in MeOH. The elution conditions were as follows: isocratic at 97% B for 4 min, gradient to 100% B from 4.1 min to 5 min, and isocratic at 100% B to 10 min. At 10.1 min, the mobile phase was reached again 97% B for 10 min to prepare the column for the next sample. The column temperature was maintained at 40 °C and controlled with the column oven (CTO-20AC). The injected sample volume was 30 µL.

Detection was performed with the LCMS-8050 triple quadrupole mass spectrometer (Shimadzu, Kyoto, Japan) equipped with the atmospheric pressure chemical ionization (APCI) source operating in the positive ion mode under the following conditions: the APCI temperature 350 °C, the desolvation line temperature 200 °C, the heating block temperature 200 °C, the nebulizer gas flow 3 L/min, and the drying gas flow 5 L/min.

To determine the optimal conditions for the chromatographic separation, the influence of the mobile phase composition (MeOH and ACN), the volume of formic acid added to the mobile phase (0.05% and 0.1%), the flow rate, the gradient elution profile, the column temperature, and the volume of the injected sample were investigated. To establish the MS/MS operating conditions, the analyte and IS standards, each at the concentration of 100 ng/mL, were separately injected into the LC-MS/MS system. For each compound, mass transitions of the most sensitive or selective precursor ions were optimized in terms of their product ions and corresponding collision energy.

### 3.5. Method Validation

The validation method was used according to the general validation criteria in terms of specificity, linearity, the limit of quantitation (LOQ), the intra-day and inter-day precision and accuracy, matrix effects, as well as stability of measurements in compliance with the main FDA guidelines for the bioanalytical method validation [31]. In addition, the dilution precision was included as the validation parameter, taking into account a possible higher concentration level of the analyte after its supplementation. Due to the lack of commercially available serum with appropriate characteristics, process optimization, calibration, and validation were performed using a representative portion of serum obtained from the individual volunteers’ blood samples that were pooled and checked for the presence of vitamin K2MK-7 after their UV irradiation to photodegrade the endogenous vitamin. After combining, the serum was mixed thoroughly to ensure its homogeneity and divided into smaller portions that were separately frozen. There are no commercially available K2MK-7 quality control (QC) samples; therefore, the aliquots of the blank serum samples were fortified with stock standards solutions to obtain low, medium, and high QC samples. During the experiments, the samples were prepared daily, except for those used to estimate the measurements stability.

To establish a calibration curve, the aliquots of the blank pooled serum sample were spiked with the sequentially increased amounts of the K2MK-7 standard and the same amount of the deuterium-labeled K2MK-7 standard used as the internal standard (IS, 10 ng/mL). Nine calibration samples were examined, including the blank sample, the zero sample (the blank sample with added IS), and seven non-zero samples in the range of 0.1 ng/mL to 1.2 ng/mL of K2MK-7. Three replicated analytical procedures were applied independently for each examined concentration level. A calibration curve was constructed by plotting the peak area ratio of K2MK-7 to IS against the known K2MK-7 concentration. The slope, intercept, and coefficient of determination (R^2^) were determined by the least squares linear regression model. To assess the quality of linearity, the statistical approach of lack of correlation was used. The quality of calibration was evaluated by the back-calculation of K2MK-7 concentrations in the calibration solutions. The lowest limit of quantification (LLOQ) was obtained on the basis of the smallest concentration of K2MK-7 that gives the CV and bias values ≤ 20%.

The limits of detection and quantification values, LOD and LOQ, respectively, were determined from the analysis of the sample chromatogram obtained for the blank plasma samples enriched with the analyte at the LLOQ level. The LOD and LOQ were considered to be the signal to the noise ratios equal to 3 and 10, respectively.

The intra- and inter-day precision and accuracy were assayed using the QC samples at three concentration levels covering the calibrating range of K2MK-7 concentrations (0.32, 0.64, and 0.96 ng/mL) and the LLOQ level (0.10 ng/mL), and evaluated by the statistical analysis. Five replicates per concentration for the independently prepared samples were analyzed three times on the same day and on five different days within three weeks. The precision was estimated using the one-way analysis of variance (ANOVA) test, and it was expressed as the coefficient of variation (CV, %). The accuracy estimation was made comparing the mean value of the obtained results to the nominal concentration level of the analyte in the control sample using the Student’s *t*-test, and it was expressed as BIAS (in %). The precision and accuracy were also tested for the pooled serum samples fortified with the standards above the concentration of the highest calibration standard. In these experiments, the samples were appropriately diluted with the blank serum to give concentrations within the calibration range. The assays were performed preparing the samples at three concentration levels (1.5, 6, and 12 ng/mL) that were diluted to 1:1, 1:8, and 1:16, respectively. The analysis of the diluted samples was performed in the set of 3 replicates for each concentration. Then, the accuracy and precision were determined.

Matrix effect (ME), recovery (RE), and process efficiency (PE) were determined using the method described in [33] by determining the area of the K2MK-7 and IS peaks in three differently prepared sets of samples, each at three concentration levels corresponding to the concentration of the QC samples. The first set was composed of standard solutions prepared in water instead of serum. The second was prepared in the aliquots of the pooled blank sample fortified with the vitamin and IS after the sample preparation. The last one was prepared in the same serum spiked with the standards prior to the extraction.

The stability of K2MK-7 was assessed for the QC samples at the low, medium, and high concentration levels with the exception of the freeze-thaw tests carried out only for the medium QC level. The stability was evaluated for the samples stored under different conditions: in the autosampler over 24 h at 15 °C, and alternatively, in the refrigerator and the freezer at 4 °C and −18 °C, respectively; exposed to light at room temperature for 1 and 2 h; and four freeze–thaw cycles from −18 °C to room temperature. Three replicates were performed under each condition. Stability was defined as <15% loss of the initial vitamin concentration. The QC samples freshly prepared and measured immediately prior to storage effect testing were used as a reference point.

### 3.6. Statistical Analysis

The Spearman’s rank correlation (rho) was used to estimate the degree of association between the K2MK-7 concentration and the age for a given gender group. The one-way analysis of variance (ANOVA) and *F*-test were used to determine the trending age groups for the given and different gender groups. To determine the significance of each Fisher coefficient (*F*), the *p*-values were used. The values were considered to be significantly different when the result of the compared parameters differed at the *p* = 0.05 significance level. The statistical analysis was performed using Excel (Microsoft Excel 2010).

## 4. Conclusions

The ample evidence for the relationship between the low vitamin K2MK-7 content in the human body and the occurrence of various dangerous diseases aroused wide interest in this compound both among scientists and average consumers following a healthy diet. As a result, there is an increasing demand for vitamin K determination and a steady increase in the number of samples required to be tested each day. To meet these expectations, in this paper, a simple, efficient, and affordable sample preparation method using the LLME approach, followed by the LC-MS/MS method for the determination of K2MK-7 in the serum samples, was developed. The method was validated according to the FDA guidelines. Then, its feasibility in evaluating K2MK-7 in real samples was tested, proving that it can be adopted as a diagnostic and preventive analytics tool. Under the optimized conditions, using 500 µL of serum and the same amount of n-hexane, the reproducibility and the accuracy were obtained in the ranges of 89–97% and 86–110%, respectively, and the limit of detection value was 0.01 ng/mL. The analysis time for K2MK-7 is about 5 min, with a total analysis time of 10 min necessary to elute more hydrophobic interfering compounds. Based on the results obtained during 2 years, we were able to statistically assess its content and distribution in the Polish population. The obtained Spearman’s rank correlation coefficients showed that the K2MK-7 values are significantly related to the age of both females and males. The higher values of the *F*-test obtained for females not only confirmed the relationship between the age and gender with the content of vitamin K, but also indicated that in the female group, there is a greater distribution of K2MK-7 values depending on age. The results will be useful for the subsequent development of K2MK-7 reference intervals in the Polish population.

## Figures and Tables

**Table 1:** Summary of the statistical analysis of the obtained results.

Effects		*F_cal_*-Value	*p*-Value	*F_tab_*-Value
Effect of solvent type on K2MK-7 peak area		48.45	1.64 × 10^−6^	3.48
Effect of EtOH adding to the extraction system on K2MK-7 peak area		129.85	1.42 × 10^−8^	3.48
Effect of ACN adding to the extraction system on K2MK-7 peak area		72.18	2.45 × 10^−7^	3.48
Effect of AA adding to the extraction system on K2MK-7 peak area		3.93	0.12	7.71
Effect of CH_2_Cl_2_ volume		1.82	0.24	5.14
Effect of CHCl_3_ volume		6.48	0.03	5.14
Effect of Et_2_O volume		2.57	0.16	5.14
Effect of CCl_4_ volume		6.01	0.04	5.14
Effect of *n*-hexane volume		83.97	4.10 × 10^−5^	5.14
Effect of age on K2MK7 concentration in female		4.18	2.18 × 10^−4^	2.05
Effect of age on K2MK7 concentration in male		3.85	5.45 × 10^−4^	2.05
Effect of sex on K2MK-7 concentration in individual age-groups:	<10	0.19	0.66	4.26
	11–20	1.26	0.27	4.06
	21–30	0.24	0.63	3.96
	31–40	1.25	0.26	3.92
	41–50	3.97	4.8 × 10^−2^	3.94
	51–60	0.14	0.71	3.96
	61–70	2.71	0.10	4.04
	>71	0.06	0.81	4.41
		*rho-value*	*p*-value	
Correlation between age and K2MK-7 concentration in female		0.23	2.34 × 10^−4^	
Correlation between age and K2MK-7 concentration in male		0.27	2.68 × 10^−5^	
Resultant correlation between age and K2MK7 concentration		0.27	2.46 × 10^−9^	

**Table 2:** Intra- and inter-day precision, accuracy, and summary of stability study for the determination of K2MK-7 in the serum samples.

Nominal Concentration (ng/mL)	Measured Concentration (mean ± SD), (ng/mL)	Imprecision (% CV)	Inaccuracy (% BIAS)
Intra-day (*n* = 5)			
0.10	0.116 ± 0.004	3.98	16.16
0.32	0.352 ± 0.022	6.40	10.10
0.64	0.728 ± 0.069	9.49	−13.80
0.96	0.939 ± 0.030	3.29	−2.12
Inter-day (*n* = 5)			
0.10	0.108 ± 0.005	4.51	8.63
0.32	0.317 ± 0.030	9.48	0.98
0.64	0.649 ± 0.072	11.04	−1.38
0.96	1.007 ± 0.073	7.22	−4.88
Autosampler at 15 °C (24 h) (*n* = 3)			
0.32	0.352 ± 0.01	2.8	−10.0
0.64	0.621 ± 0.084	13.52	3.06
0.96	1.031 ± 0.052	5.04	−12.81
Refrigerator at 4 °C (24 h) (*n* = 3)			
0.32	0.360 ± 0.06	13.76	12.50
0.64	0.622 ± 0.06	8.59	2.82
0.96	1.014 ± 0.046	4.44	−5.62
Freezer at −18 °C (24 h) (*n* = 3)			
0.32	0.325 ± 0.042	12.92	1.56
0.64	0.619 ± 0.033	5.33	3.28
0.96	1.014 ± 0.046	5.80	6.66
Exposed to light at room temp. (1 h) (*n* = 3)			
0.32	0.167 ± 0.004	2.39	47.81
0.64	0.250 ± 0.188	75.08	60.88
0.96	0.344 ± 0.240	69.79	64.18
Exposed to light at room temp. (2 h) (*n* = 3)			
0.32	0.125 ± 0.060	47.83	60.80
0.64	0.299 ± 0.092	30.91	53.19
0.96	0.508 ± 0.12	23.60	47.03
Freeze-thaw cycles (cycle number) (*n* = 3)			
0.64 (1)	0.600 ± 0.014	2.33	6.20
0.64 (2)	0.716 ± 0.041	5.74	−11.94
0.64 (3)	0.657 ± 0.058	8.83	−2.65
0.64 (4)	0.379 ± 0.052	13.71	40.66

**Table 3:** Summary of matrix effect (ME), recovery (RE), and process efficiency (PE) studies for K2MK-7 and IS at three levels of vitamin concentrations covering the calibration range for three differently prepared sets of samples: set 1 is samples of standard solutions prepared in water instead of serum, sets 2 and 3 are pooled blank serum samples that were fortified with standard solutions after extraction (set 2) and before extraction (set 3).

Nominal Concentration (ng/mL)	Mean Peak Area (% CV)						ME (%)		RE (%)		PE (%)	
	K2MK-7			IS								
	Set 1	Set 2	Set 3	Set 1	Set 2	Set 3	K2MK-7	IS	K2MK-7	IS	K2MK-7	IS
0.32	16,954 (5.21)	19,281 (1.34)	15,280 (9.54)	35,768 (2.55)	38,317 (2.25)	32,313 (9.14)	113.73	107.13	79.25	84.33	90.13	90.34
0.64	33,288 (10.87)	36,584 (9.12)	30,011 (9.20)	32,235 (7.76)	36,701 (1.28)	31,833 (15.70)	109.90	113.85	82.03	86.74	90.15	98.75
0.96	48,747 (4.08)	49,094 (0.72)	41,721 (2.58)	33,729 (11.95)	34,329 (2.13)	30,039 (10.51)	100.71	101.78	84.98	87.50	85.59	89.06

## Data Availability

Not applicable.

## Supplementary Materials

The following supporting information can be downloaded at: https://www.mdpi.com/article/10.3390/molecules28186523/s1, Figure S1: Calibration curve with marked confidence intervals at the confidence level 0.95.; Figure S2. Exemplary chromatograms obtained during the optimization of sample preparation procedure using different extraction solvents, i.e. CCl_4_ (A), CH_2_Cl_2_ (B), CHCl_3_ (C), Et_2_O (D), and n-hexane (E), respectively. Table S1: Raw data for calibration curve together with statistical analysis; Table S2. The lack-of-fit test; Table S3. The influence of the number of extraction cycles on the surface area of the vitamin K2MK-7 peak extracted using 500 and 250 µL of *n*-hexane, respectively; Table S4. Results of comparative studies.

## Sample Availability

Samples of the compounds are not available from the authors.
