# Supplementary material for: Development, Validation, and Two-Year Application of Rapid and Simple LC-MS/MS-Based Method for the Determination of K2MK-7 in Blood Samples
Source: Molecules. 2023 Sep 8;28(18):6523. doi: 10.3390/molecules28186523 (PMC10535264; doi:10.3390/molecules28186523)
Supplement: Supplementary file 1 [file molecules-28-06523-s001.zip › molecules-2576855-supplementary.pdf]

Supplementary Materials

Development, Validation, and Two-Year Application of Rapid and Simple LC-MS/MS-Based Method for the Determination of K2MK-7 in Blood Samples

Łukasz Paprotny , Dorota Szewczak , Iryna Bryshten and Dorota Wianowska

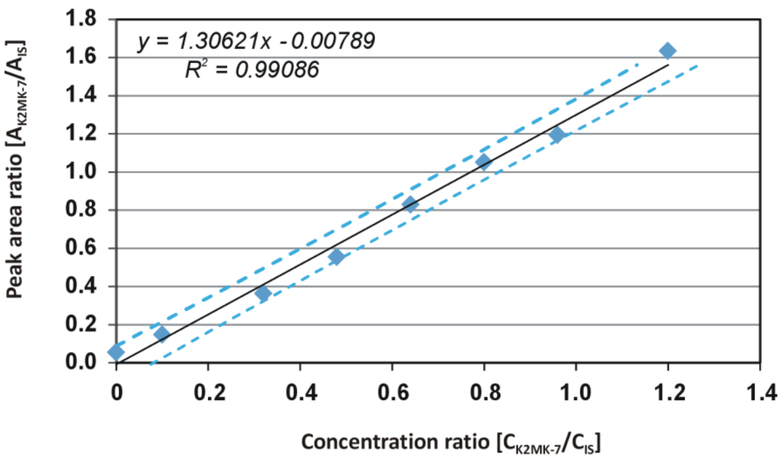

Figure S1. Calibration curve with marked confidence intervals at the confidence level 0.95.

Table S1. Raw data for calibration curve together with statistical analysis

n	Xi	Yi-1	Yi-2	Yi-3	Yi	XiYi			Xi^2	Yi^2	Xi-X_	(Xi-X_)^2	Yi-Y_	(Yi-Y_)^2	(Xi-X_)(Yi-Y_)	di	di^2
1	0	0,0528 596	0,0528 6	0,0528 6	0,0529	0	0	0	0	0,002794 137	- 0,562 5	0,3164 06	- 0,6739 9	0,4542 61	0,379119	0,06075 389	0,0036 91
2	0,1	0,1453 76	0,1520 94	0,1401 87	0,1459	0,0145 89	0,0152 09	0,0140 187	0,01	0,021282 628	- 0,462 5	0,2139 06	- 0,5809 6	0,3375 18	0,268695	0,02315 901	0,0005 36
3	0,32	0,3872 64	0,2862 61	0,4123 77	0,3620	0,1158 3	0,0916 04	0,1319 606	0,102 4	0,131020 35	- 0,242 5	0,0588 06	- 0,3648 8	0,1331 38	0,088484	- 0,04812 54	0,0023 16
4	0,48	0,5982 49	-	0,5073 98	0,5528	0,2653 55	#ARG !	0,2435 51	0,230 4	0,305613 822	- 0,082 5	0,0068 06	- 0,1740 3	0,0302 85	0,014357	- 0,06626 27	0,0043 91
5	0,64	0,7467 53	0,9742 28	0,7628 35	0,8279	0,5298 81	0,6235 06	0,4882 144	0,409 6	0,685482 436	0,077 5	0,0060 06	0,1010 9	0,0102 19	0,007834	- 0,00014 11	1,99E- 08
6	0,8	1,0282 7	1,0756 2	1,0422	1,0487	0,8389 57	0,8604 96	0,8337 6	0,64	1,099764 699	0,237 5	0,0564 06	0,3218 48	0,1035 86	0,076439	0,01162 342	0,0001 35
7	0,96	1,4157 7	1,0811 8	1,0801 2	1,1924	1,1446 62	1,0379 33	1,0369 152	0,921 6	1,421714 421	0,397 5	0,1580 06	0,4655 08	0,2166 98	0,185039	- 0,05371 01	0,0028 85
8	1,2	1,7325	1,5274 6	1,6368 2	1,6323	1,9587 12	1,8329 52	1,9641 84	1,44	2,664272 708	0,637 5	0,4064 06	0,9054 11	0,8197 7	0,5772	0,07270 298	0,0052 86
								0,5528 235									
								0,0642 414									
	X_				Y_												
Średnia	*0,5625*				*0,726848 513*	*0,6084 98*		*11,620 59*	*0,469 25*	*0,791493 15*							
n	SXi				SYi	SXiYi			S(Xi^2)	S(Yi^2)					S(Xi-X_)(Yi-Y_)		S(di^2)
**8**	**4,5**				**5,814788 1**	**4,8679 86**			**3,754**	**6,331945 2**		**1,2227 5**		**2,1054 75**	**1,597168**		**0,0192 4**

n	a nS(XiYi)-SXiSYi	b S(Xi^2)Syi-(SXiYi)Sxi	S_y S(di^2)/(n-2)	D S(Xi^2)*n	S_a^2 (S_{y^2}n)/D	S_b^2 (S_{y^2}S(Xi^2))/D	S_a Ö(Sa^2)	S_b Ö(Sb^2)	R^2 (S(Xi-X_)(Yi-Y_))^2
1	12,77734	- 0,0772 22	0,003207	30,032	0,0256530 66	0,012038	0,051 21	0,035 08	2,550944237
3	nS(Xi^2)-(Sxi)^2	nS(Xi^2)-(Sxi)^2	Ö()	Sxi*Sxi					S(Xi-X_)^2S(Yi-Y_)^2
4	9,782	9,782	0,056627	20,25					2,574469702
6	1,306209	- 0,0078 94		9,782	0,0026224 77	0,001231			0,990862015

y=ax+b

a	*przedział ufności*	*b*	*przedział ufności*
1,3062 094	0,1060 05	-0,007894	0,0726153 27

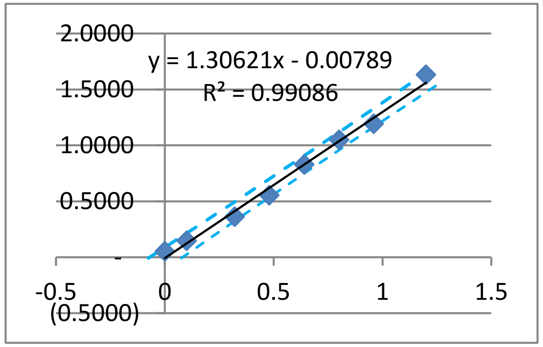

**Xi**	**Xical**	**% Bias**
0	0,046 51	#DZIEL/ 0!
0,1	0,117 73	17,73
0,32	0,283 16	-11,51
0,48	0,429 27	-10,57
0,64	0,639 89	-0,02
0,8	0,808 90	1,11

linia	0	- 0,0078 94
	1,2	1,5595 57

no	x	y		-	+
0	0	-0,00789	0,037	-0,0757	0,075699
1	0,06	0,070478	0,036	-0,01354	0,133536
2	0,12	0,148851	0,035	0,048498	0,191502
3	0,18	0,227223	0,034	0,110391	0,249609
4	0,24	0,305596	0,033	0,172133	0,307867

0,96	0,918<br>88	-4,28
1,2	1,255<br>66	4,64

						n-2
				**poziom ufności**	0,95	*t* **2,07**
				**przedział ufności**		
**n**	**Yi**	**Xi**	**Sx**	**Xi**		
1	0,0528<br>6	0	0,036	-0,07	0,07	
2	0,1458<br>86	0,1	0,034	0,03	0,17	
3	0,3619<br>67	0,32	0,031	0,26	0,38	
4	0,5528<br>24	0,48	0,030	0,42	0,54	
5	0,8279<br>39	0,64	0,030	0,58	0,70	
6	1,0486<br>97	0,8	0,031	0,74	0,86	
7	1,1923<br>57	0,96	0,033	0,89	1,03	
8	1,6323	1,2	0,040	1,12	1,28	

5	0,3	0,383969	0,032	0,23371	0,36629
6	0,36	0,462341	0,031	0,295112	0,424888
7	0,42	0,540714	0,031	0,356325	0,483675
8	0,48	0,619086	0,030	0,41734	0,54266
9	0,54	0,697459	0,030	0,478147	0,601853
10	0,6	0,775831	0,030	0,538737	0,661263
11	0,66	0,854204	0,029	0,599104	0,720896
12	0,72	0,932576	0,029	0,659245	0,780755
13	0,78	1,010949	0,029	0,719157	0,840843
14	0,84	1,089322	0,030	0,778841	0,901159
15	0,9	1,167694	0,030	0,838301	0,961699
16	0,96	1,246067	0,030	0,897543	1,022457
17	1,02	1,324439	0,031	0,956575	1,083425
18	1,08	1,402812	0,031	1,015406	1,144594
19	1,14	1,481184	0,032	1,074046	1,205954
20	1,2	1,559557	0,033	1,132508	1,267492

Table S2. The lack-of-fit test

H_0 There is no lack of fit

H_A There is lack of fit

Concentration	Signal 1	Signal 2	Signal 3	Mean signal	Signal_calc (au)	(mean-calculated)^2	MSS (LoF)	(Signal1(i)-mean_signal(i))^2	(Signal2(i)-mean_signal(i))^2	(Signal3(i)-mean_signal(i))^2	MSS (error)
0	0,0520775	0,0672389	0,052852	0,0574	-0,0079	0,004262	0,00515	0,0000282	0,0000970	0,0000206	0,010184
0,1	0,145376	0,152094	0,140187	0,1459	0,1227	0,000536		0,0000003	0,0000385	0,0000325	
0,32	0,387264	0,286261	0,412377	0,3620	0,4101	0,002316		0,0006399	0,0057314	0,0025411	
0,48	0,598249	0,378097	0,507398	0,4946	0,6191	0,015501		0,0107470	0,0135686	0,0001643	
0,64	0,746753	0,974228	0,762835	0,8279	0,8281	0,000000		0,0065911	0,0214006	0,0042385	
0,8	1,02827	1,07562	1,0422	1,0487	1,0371	0,000135		0,0004172	0,0007249	0,0000422	
0,96	1,41577	1,08118	1,08012	1,1924	1,2461	0,002885		0,0499135	0,0123603	0,0125971	
1,2	1,7325	1,52746	1,63682	1,6323	1,5596	0,005286		0,0100481	0,0109830	0,0000208	

slope	1,3062094
intercept	-0,0078943
n	8
p	3
DoF (numerator)=	7
DoF (denominator)=	18
F_tab=	2,58
F_calc=	0,790

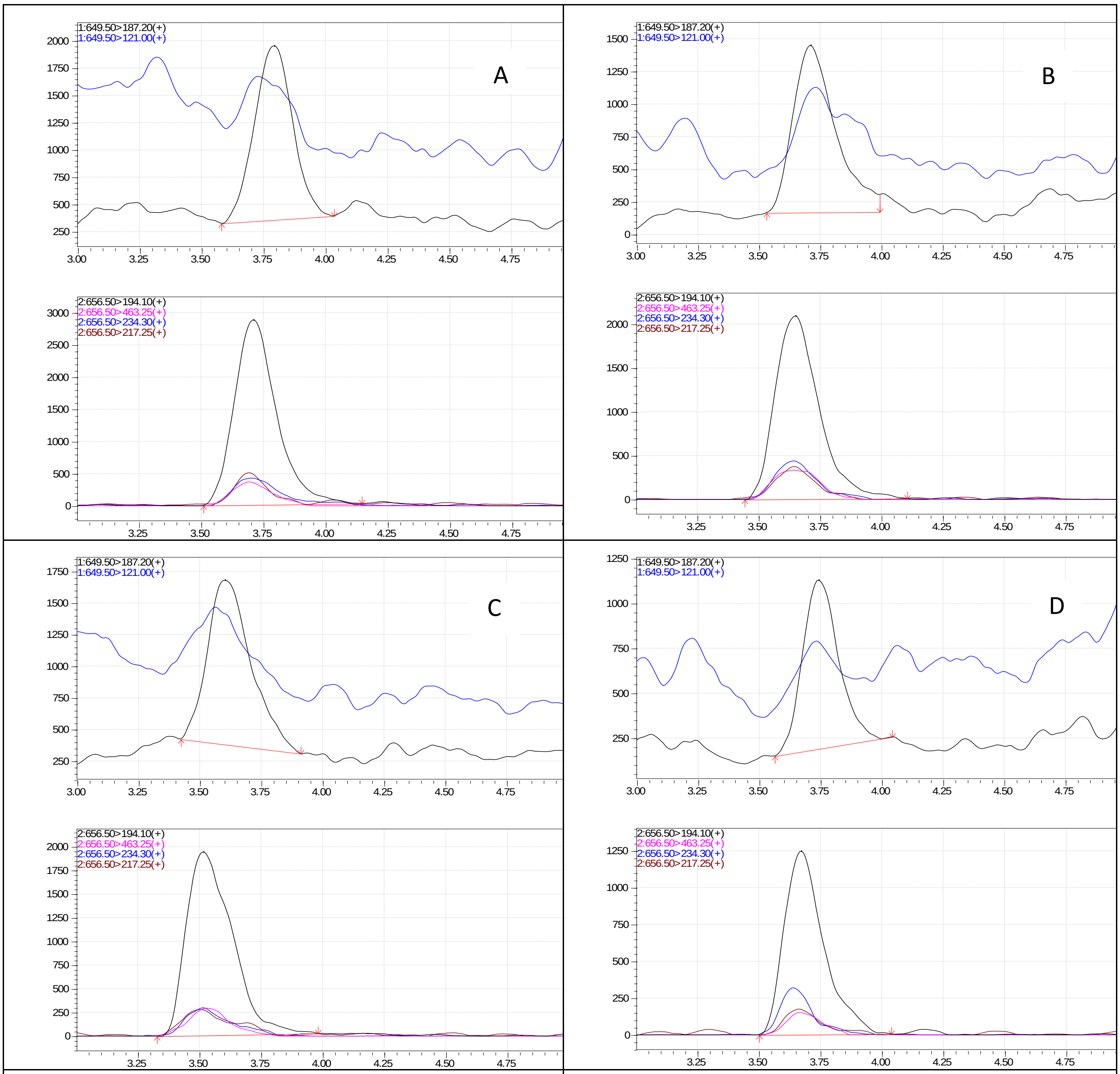

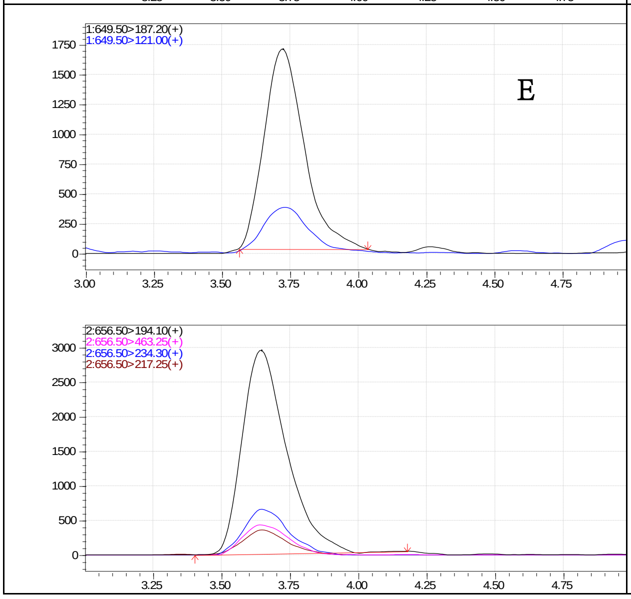

Figure S2. Exemplary chromatograms obtained during the optimization of sample preparation procedure using different extraction solvents, i.e. CCl_4 (A), CH_2Cl_2 (B), $CHCl_3$ (C), Et_2O (D), and n-hexane (E), respectively

Table S3. The influence of the number of extraction cycles on the surface area of the vitamin K2MK-7 peak extracted using 500 and 250 μL of n-hexane, respectively

Cycle number	500 μL	250 μL
1	31194 ± 674	19071 ± 622
2	1940 ± 483	7247 ± 1141
3	n.d.	6371 ± 701
Total	33134 ± 1157	32689 ± 2464

Table S4. Results of comparative studies

Sample number	Lab 1	Lab 2
1	1,3412	1,1467
2	2,6195	2,3838
3	0,2842	0,2473
4	0,4484	0,4874
5	0,4441	0,3819
6	0,1461	0,1242
7	3,6302	3,0494
8	0,1585	0,1426
9	0,3492	0,3212
10	3,2486	3,6501
11	0,1400	0,1246
12	11,6607	10,4946
13	9,8915	8,9024
14	0,5110	0,4343
15	0,1012	0,1205
16	0,2309	0,1986
17	1,1451	0,9963
18	1,9074	1,8120
19	0,4140	0,3560
20	0,3700	0,3145
21	0,1470	0,1279
22	0,8100	0,7290
23	0,2026	0,1732
24	1,4940	1,3595
25	2,9381	3,3771
26	2,7799	2,5575
27	0,6037	0,5192
28	2,3655	2,0107
29	0,4067	0,3416
30	0,1474	0,1327
31	0,2588	0,2381
32	0,3824	0,4297

33	0,0815	0,0725
34	0,1424	0,1282
35	2,1365	1,9229
36	0,3816	0,3244
37	0,7482	0,6285
38	0,6977	0,8113
39	0,3426	0,3938
40	0,1617	0,1536
41	3,6728	3,1586
42	2,7141	3,1930
43	2,6850	2,3360
44	1,9942	1,7948
45	1,5802	1,3511
46	0,2022	0,2222
47	1,7170	1,4938
48	2,2546	2,0742
49	0,1250	0,1075
50	3,1480	3,7035
